# Lymph node pooling: a feasible and efficient method of lymph node molecular staging in colorectal carcinoma

**DOI:** 10.1186/s12967-016-1114-3

**Published:** 2017-01-14

**Authors:** Natalia Rakislova, Carla Montironi, Iban Aldecoa, Eva Fernandez, Josep Antoni Bombi, Mireya Jimeno, Francesc Balaguer, Maria Pellise, Antoni Castells, Miriam Cuatrecasas

**Affiliations:** 1 Pathology Department-Centre de Diagnòstic Biomèdic (CDB), Hospital Clínic, University of Barcelona, Escala 3, Planta 5, 08036 Barcelona, Spain; 2 Gastroenterology Department, Hospital Clinic, CIBERehd, IDIBAPS, University of Barcelona, Barcelona, Spain; 3 Tumor Bank, Biobanc Clinic-IDIBAPS and Xarxa de Bancs de Tumors de Catalunya (XBTC), Barcelona, Spain

**Keywords:** Colon cancer, Lymph node, Pooling, Staging, Molecular, OSNA, Total tumor load

## Abstract

**Background:**

Pathologic lymph node staging is becoming a deficient method in the demanding molecular era. Nevertheless, the use of more sensitive molecular analysis for nodal staging is hampered by its high costs and extensive time requirements. Our aim is to take a step forward in colon cancer (CC) lymph node (LN) pathology diagnosis by proposing a feasible and efficient molecular method in routine practice using reverse transcription loop-mediated isothermal amplification (RT-LAMP).

**Results:**

Molecular detection of tumor cytokeratin 19 (CK19) mRNA with RT-LAMP was performed in 3206 LNs from 188 CC patients using two methods: individual analysis of 1449 LNs from 102 patients (individual cohort), and pooled LN analysis of 1757 LNs from 86 patients (pooling cohort). A median of 13 LNs (IQR 10–18) per patient were harvested in the individual cohort, and 18 LNs (IQR 13–25) per patient in the pooling cohort (p ≤ 0.001). The median of molecular assays performed in the pooling cohort was 2 per patient (IQR 1–3), saving a median of 16 assays/patient. The number of molecular assays performed in the individual cohort was 13 (IQR 10–18), corresponding to the number of LNs to be analyzed. The sensitivity and specificity of the pooling method for LN involvement (assessed by hematoxylin and eosin) were 88.9% (95% CI 56.5–98.0) and 79.2% (95% CI 68.9–86.8), respectively; concordance, 80.2%; PPV, 33.3%; NPV, 98.4%. The individual method had 100% sensitivity (95% CI 72.2–100), 44.6% specificity (95% CI 34.8–54.7), 50% concordance, 16.4% PPV, and 100% NPV. The amount of tumor burden detected in all LNs of a case, or total tumor load (TTL) was similar in both cohorts (p = 0.228).

**Conclusions:**

LN pooling makes it possible to analyze a high number of LNs from surgical colectomies with few molecular tests per patient. This approach enables a feasible means to integrate LN molecular analysis from CC specimens into pathology diagnosis and provides a more accurate LN pathological staging with potential prognostic implications.

## Background

Colorectal cancer (CRC) is one of the most prevalent malignancies and the second cause of death from cancer in developed countries [1]. Importantly, up to 25% of curative-intended surgically treated CRC patients develop local recurrence or distant metastases within 5 years of surgery [2–5]. Overall and disease-free survival rates after curative surgical treatment depend on the T and N stages of disease [6–9], but lymph node (LN) status is the most powerful prognostic factor [4, 10–12]. Thus, pathological analysis of at least 12 LNs from surgical colectomies is required to ensure a reliable pN0 stage and is among the key quality measures for CRC care [13]. Moreover, the accuracy and predictive value of assigning stage II are directly proportional to the number of LNs examined [14].

Molecular LN assessment to detect tumor burden is far more sensitive than the standard hematoxylin and eosin (H&E) analysis [15–19]. Whereas molecular methods can analyze the entire LN, <0.5% of the LN is histologically examined. Thus, pathological nodal staging (pN) may not be representative of the LN status and can lead to false negative diagnoses [16, 19, 20].

The use of more sensitive molecular methods of LN staging, rather than H&E, would be strongly recommended in stage I–II CRC to help identify those patients at risk of recurrence [16–21]. In addition, recently implemented CRC screening programs result in earlier stage tumor detection [22, 23]. In this setting, a more accurate diagnosis and staging is mandatory. However, given the high prevalence of CRC and the high number of LNs to be analyzed, systematic molecular LN analysis or additional diagnostic methods beyond routine H&E are far from being incorporated into pathological diagnosis, mostly because of the high cost of molecular techniques and the supplementary workload.

The molecular assay One Step Nucleic Acid Amplification (OSNA; Sysmex Corporation, Kobe, Japan) is an automated, quantitative and high-sensitive assay for detection of cytokeratin 19 (CK19) messenger RNA (mRNA). It uses the reverse transcription loop-mediated isothermal amplification method (RT-LAMP) to amplify CK19 mRNA from LN tissue lysates. The assay has been validated for breast and CRC lymph node assessment, providing results comparable to extensive histological and immunohistochemical (IHC) LN analyses [15, 24–26]. Additionally, it makes it possible to obtain the total tumor load (TTL), defined as the sum of CK19 mRNA copies/µL from each positive LN. The TTL corresponds to the amount of tumor burden present in all the LNs of a surgical specimen [26–28].

In this study we compared two methods of LN molecular analysis, the individual analysis of each LN, and the new approach consisting in pooling several LNs into microcentrifuge tubes for analysis (onwards pooling method). We aimed to demonstrate the efficiency of the new procedure in the analysis of colon cancer (CC) LNs in terms of time spent in the molecular analysis, as well as the feasibility of introducing this approach into pathology daily practice. Molecular LN staging provides a practical approach for a more accurate diagnosis, which would enable the use of molecular data in CRC patient’s therapeutic management.

## Results

### Patients and lymph node characteristics

A total of 3206 LNs from 188 colectomy specimens of surgically treated CC patients were analyzed by OSNA; 1757 LNs from 86 patients were analyzed using the pooling approach (pooling cohort), and 1449 LNs from 102 patients were analyzed using individual LN analysis (individual cohort). The pooling cohort had more high grade cases (p < 0.001), with higher rates of vascular and perineural invasion (p = 0.045 and p = 0.013, respectively) and more advanced pT stages (p = 0.004). In addition, it also harbored higher yields of fresh and total LNs (p < 0.001). The differences among the two cohorts are due to the fact that the pooling cohort is more recent and all tumors were included irrespective of the gross appearance of LNs. The individual cohort is older and cases with grossly positive LNs cases were discarded, in an effort to obtain pN0 cases for molecular correlation. All positive LN cases with H&E corresponded to histological findings. Tables 1 and 2 show baseline characteristics of patients included in the study.

**Table 1:** Patient’s demographics and specimen characteristics from the two cohorts

Clinical parameter	n (%)/median (IQR)		*p* value
	OSNA pooling cohort	OSNA individual cohort	
Cases	86 (100)	102 (100)	
Gender			0.974
Male	55 (64.0)	65 (63.7)	
Female	31 (36.0)	37 (36.3)	
Age (years)	70.0 (63.0–79.0)	65.5 (59.0–72.0)	0.0635
Tumor characteristics			
Size (cm)	3.0 (1.7–4.5)	2.5 (1.1–3.7)	0.992
Location			0.090
Right colon	39 (45.3)	51 (50.0)	
Flexures/transverse	1 (1.2)	7 (6.9)	
Left colon	46 (53.5)	44 (43.1)	
Gross configuration			0.821
Polypoid	57 (66.3)	66 (64.7)	
Ulcerated	29 (33.7)	36 (35.3)	
Grade			<0.001
High	49 (57.0)	26 (25.5)	
Low	37 (43.0)	76 (74.5)	
Type of tumor			0.118
Adenoma	6 (7.0)	8 (7.8)	
Carcinoma in situ	6 (7.0)	17 (16.7)	
Adenocarcinoma	74 (86.0)	77 (75.5)	
Budding			0.650
Absence	13 (17.6)	14 (18.7)	
Low grade	47 (63.5)	51 (68.0)	
High grade	14 (18.9)	10 (13.3)	
Vascular invasion			0.045
No	65 (75.6)	91 (89.2)	
Yes	21 (24.4)	11 (10.8)	
Perineural invasion			0.013
No	76 (88.4)	98 (96.1)	
Yes	10 (11.6)	4 (3.9)	
Tumor deposits			0.165
No	81 (94.2)	100 (98.0)	
Yes	5 (5.8)	2 (2.0)	
pT			0.004
pT0	6 (7.0)	8 (7.8)	
pTis	6 (7.0)	17 (16.7)	
pT1	9 (10.4)	26 (25.6)	
pT2	16 (18.6)	19 (18.6)	
pT3	32 (37.2)	24 (23.5)	
pT4a	17 (19.8)	8 (7.8)	
Lymph nodes			
Total LN	1992 (100)	1828 (100)	
Fresh LN	1757 (88.2)	1449 (79.3)	
FFPE LN	235 (11.8)	379 (20.7)	

*IQR* interquartile range, *LN* lymph node, *FFPE* formalin-fixed paraffin-embedded, p-value: using t test for continuous variables and Chi square for categorical variables

**Table 2:** Lymph node characteristics and OSNA results

	Median (IQR)		p-value
	OSNA pooling cohort	OSNA individual cohort	
Fresh LN	18.0 (13.0–25.0)	13.0 (10.0–18.0)	<0.001
FFPE LN	1.0 (0.0–5.0)	3.0 (2.0–5.0)	<0.001
Total LN	20.5 (17.0–27.0)	17.0 (13.0–22.0)	<0.001
H&E results (pN)			
pN0	77 (89.5)	92 (90.2)	0.588
Positive	9 (10.5)	10 (9.8)	
pN1a	3 (3.5)	5 (4.9)	
pN1b	3 (3.5)	3 (2.9)	
pN1c	0	0	
pN2a	1 (1.2)	2 (2.0)	
pN2b	2 (2.3)	0	
OSNA results			
Positive	24 (27.9)	61 (59.8)	<0.001
Negative	62 (72.1)	41 (40.2)	
TTL of OSNA positive cases	5485 (855–26,350)	1940 (920–7400)	0.228
OSNA tests	2.0 (1.0–3.0)	13.0 (10.0–18.0)	<0.001
OSNA tests saved	16 (12–22)	*–*	

*IQR* interquartile range, *FFPE* formalin-fixed, paraffin embedded, *LN* lymph node

All primary tumors resulted positive for CK19 immunohistochemistry.

### Comparison of OSNA and H&E results

In the pooling cohort, 16 out of 77 (20.8%) cases that were pN0 with H&E, resulted positive with OSNA. In contrast, only one of the 9 cases with positive LNs observed with H&E was OSNA negative (11.1%). Therefore, at patient level, the sensitivity and specificity of the OSNA-pooling method compared to H&E were, respectively, 88.9% (95% CI 56.5–98.0) and 79.2% (95% CI 68.9–86.8), with a concordance of 80.2% (Table 2). The OSNA-pooling PPV and NPV were 33.3 and 98.4%, respectively.

In the individual cohort, all 10 positive H&E cases were also OSNA positive. Among 92 H&E pN0 patients, 51 (55.4%) were positive with the OSNA analysis. The sensitivity and specificity of the individual LN-OSNA analysis with respect to H&E were, respectively, 100% (95% CI 72.2–100) and 44.6% (95% CI 34.8–54.7), with a concordance of 50%, PPV of 16.4%, and NPV of 100%.

Overall, the OSNA analysis is very sensitive, and a negative result is highly reliable. On the other hand, the pooling method has a better concordance with the H&E status, mainly as it harbors a much higher PPV.

### Molecular assays and time saved

The median of LNs harvested per patient and analyzed by OSNA in the pooling cohort was 18 (13–25). All LNs were put into a median of 2 tubes (1–3) per case, saving 16 (12–22) molecular tests per case. On the other hand, the median fresh LNs harvested in the individual cohort was 13 (10–18), corresponding to the number of molecular tests performed in this group.

Additionally, the time spent in the process of molecular analysis was greatly reduced. Each run of the RD-100i system analyzes 4 tubes simultaneously in 30–40 min, including LN tissue preparation and analysis. With the pooling method a median of two tubes were collected per patient. Two patients could be analyzed at each run of the OSNA assay in 30–40 min, compared to 135–180 min spent per patient to analyze a median of 18 tubes/patient if the LN individual analysis was to be used. Given the reduction of a median of 16 tubes per patient, the time saved in the molecular tests would be 120–160 min per patient.

### Total tumor load (TTL)

The median TTL of positive cases form the pooling cohort was 5485 CK19 mRNA copies/µL (855; 26,350), and in the individual cohort it was 1940 copies/µL (920; 7400), with no significant differences between both groups (p = 0.228). In each group, there was no significant correlation between the number of histologically positive LN and the TTL, or between the number of LN analyzed and the TTL.

Among pN0 cases, in the pooling group there were 16/77 (20.7%) pN0 patients with positive OSNA results with a median TTL of 1550 copies/µL (IQR 465–6035). In the individual cohort, 51/92 (55.4%) pN0 patients resulted OSNA positive, with a median TTL of 1540 copies/µL (IQR 620–3600). The TTLs of H&E pN0 cases from both groups were similar (p = 0.837).

### Analysis of the pooling method

**Fig. 1:**
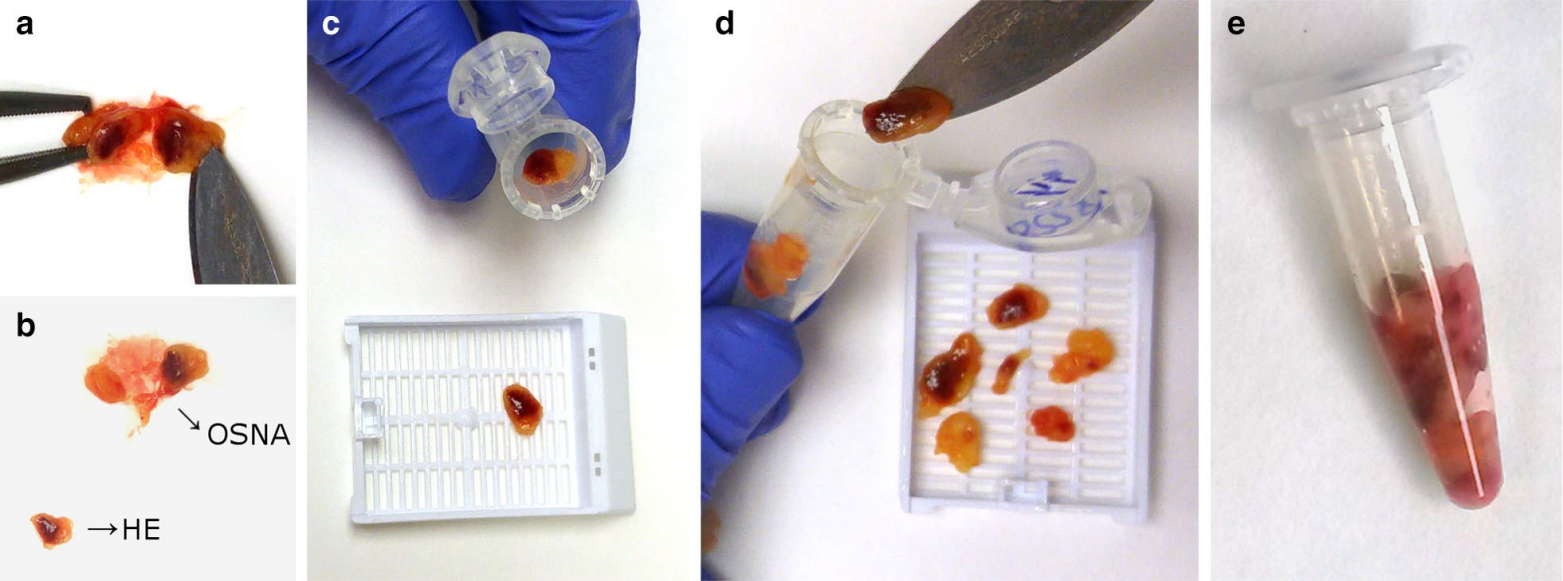
Lymph node processing for OSNA pooling and individual analysis. First two steps (**a**, **b**) are common for both cohorts. **a** Lymph nodes freshly dissected from the mesocolon fat are cut in half; **b** a 1 mm central slice is put in a cassette and used for FFPE and H&E analysis. The rest of the lymph node is put into a microcentrifuge tube for ulterior OSNA analysis; **c** Individual OSNA analysis: After the process of **a**, **b**, each central lymph node slice is individually put in a cassette for FFPE and H&E analysis. The rest of the lymph node is put into a microcentrifuge tube for individual OSNA analysis. **d** Pooling analysis: Lymph nodes are identically processed and put together into a microcentrifuge tube and a cassette for OSNA and H&E analysis, respectively. **e** Microcentrifuge tube containing multiple lymph nodes (pool) for simultaneous OSNA analysis

In 44 of the 86 cases from the pooling cohort, all LN from each colectomy specimen were collected in one tube. In the other 42 cases, all LN were collected in more than one tube; i.e. 2–7 tubes. We used the latter 42 cases to perform a validation assessment in order to evaluate whether the TTL obtained from the sum of each of the tubes was equivalent to the TTL resulting from the analysis of the content of all tubes together in a single tube (Fig. 1; Table 3).

**Table 3:** Pooling method validation approach

Analysis of a mixture of each pool in a single tube	Analysis of tubes (pools) separately		
	Positive	Negative	Total
Positive	10	2	12
Negative	2	28	30
Total	12	30	42

Correspondence of TTL values with two different analysis

Our results confirmed that the TTL resulting from the sum of the analysis of each tube separately was equivalent to the TTL obtained from the analysis of the content of all tubes mixed into a separate tube, with a sensitivity of 83.3% (95% CI 50.9–97.1), a specificity of 93.3% (95% CI 76.5–98.8), an agreement of 90.5% (95% CI 76.5–96.9), and a substantial correlation (k = 0.756). Discordant cases corresponded to very low TTL values, close to the positivity of the test of 250 copies/uL, which could be attributed to pipetting differences.

## Discussion

In this study, we used a highly sensitive molecular assay for the detection of tumor burden in a large number of LNs from CC patients. We showed that the pooling strategy is an efficient and rapid method that makes it possible to perform molecular analysis of multiple LNs at the same time, saving time and laboratory resources. This method can be easily introduced into the daily practice of pathology departments.

The need to process CRC lymph nodes using more sensitive methods than H&E has been unmet so far, given the evidence of an increased risk of recurrence and poor survival associated with the presence of undetected tumor burden with H&E in regional LNs of CRC patients [16–21]. It is time for an upgrade of the classical H&E LN diagnosis and staging, which is becoming obsolete due to its low sensitivity in detecting the presence of tumor burden within LNs, mostly as a result of sampling bias and the limited amount of LN tissue analyzed [5, 16–20, 29]. As has been stated in various studies and meta-analyses, this problem could be solved by using molecular methods [16–21].

Although stage I–II CRC patients without nodal invasion or distant metastases have a 93% 5-year survival rate, 15–20% of patients histologically staged as pN0 may recur within 5 years of curative-intended surgery [5, 12, 13, 30, 31]. The importance of a precise pathological nodal staging is critical in determining the most suitable treatment and identifying those individuals at risk of recurrence [32, 33].

The OSNA assay is an automated, quantitative, rapid, and standardized method used in pathology departments for breast sentinel LN analysis [27, 34–36]. Authors such as Castellano et al. [36] showed that it can provide useful data to decide to perform or spare axillary dissection intraoperatively. Its main advantages are that LNs can be entirely analyzed, and that it allows the use of the TTL to quantify the total amount of tumor burden present in all LNs from a surgical specimen.

We propose the use of LN pooling for the assessment of molecular LN staging in CC as a practical and novel method of multiple LN analysis. In our hands, the OSNA pooling analysis yielded better correlation with H&E LN status than the individual approach; it showed a higher PPV than the individual cohort (33.3 vs 16.4%), with similar NPV (98.4 vs 100%). The concordance between the OSNA pooling method and the H&E LN status was above 80%.

OSNA positivity rates of pN0 CRC patients are variable in previous reported studies [15, 26, 28, 37–41]. In our series, among pN0 cases, OSNA detected tumor burden in LNs of more than 50% cases from the individual cohort, with 20.8% being positive in the pooling group. The latter results were similar to other published studies [15, 26, 28, 37–41]. Croner and colleagues analyzed 1594 LNs from 103 patients and found 25.2% OSNA positivity. In their study, they performed the same LN workout as in our work [28]. Güller et al. [37] analyzed 313 LNs from 22 patients with only 15.3% OSNA positive pN0 patients. They used only half of the LN for OSNA analysis and the other half for extensive H&E and IHC workout. In addition, LNs smaller than 3 mm were not analyzed by OSNA. Similar results were obtained by Yamamoto et al. [26] with an upstaging rate of 11.3% in 1925 LNs from 124 pN0 patients, analyzing with OSNA only 44.5% of all dissected LNs and also using half of the LN for molecular analysis. They also found that the molecular positivity rate increased with the pT stage, from 2% in stage I to 25% in stage IIC. The importance of the sampling bias is reflected in these studies, with higher positivity rates obtained with the increase of the amount of LN tissue and the number of LN analyzed.

In LN molecular staging, tumor load quantification can become an objective tool that could be used to stratify the risk of an individual patient. TTL values from OSNA positive cases were 5485 copies/µL in the pooling cohort and 1940 copies/µL in the individual cohort, being lower in pN0 patients from both cohorts, i.e. 1540 and 1550 copies/µL, respectively. Furthermore, Yamamoto et al. [26] found an identical TTL amount of 1550 copies/µL in pN0 patients. They also observed an increment in the TTL with the increase in the number of positive LNs, rising to 24,050 copies/µL in pN1 patients and to 90,600 copies/µL in pN2 patients. Hence, TTL represents a continuous value of the amount of tumor burden present in the LNs of a given patient, which may be more accurate than the number of positive LNs [26, 27, 42]. Accordingly, OSNA studies in breast sentinel LNs demonstrated that the TTL held higher correlation with the prediction of additional axillary metastases than the number of positive sentinel LNs [27, 42].

Other authors used a similar model with qRT-PCR and found CEA mRNA levels in LNs of stage II CRC patients as predictors of prognosis [43]. Yet, using GUCY2C RT-qPCR, Hyslop found occult tumor burden in 87.5% of pN0 LNs, although only 20.9% of patients developed recurrent disease [17]. In our study, the rate of pN0 cases that were OSNA positive in the pooling cohort was 20.8%, similar to the recurrence rates in stage II CRC patients published in the literature [5, 12, 13, 30, 31].

Establishing cutoff values that hold biological significance is mandatory in molecular assays. In CRC, Croner tentatively stated a TTL of 4250 copies obtained with OSNA as determinant of macrometastasis [28]. In breast sentinel LN models, TTL values of 15,000 to 1.2 × 10^5^ copies of CK19 mRNA have been established as the cutoff to help clinicians decide on axillary LN dissection [27, 42]. Some authors have designed nomograms correlating different predictive values, as the clinical significance of the TTL may be modulated by other pathological factors [44]. Although our results are preliminary and not yet validated, we observed that TTL values of at least 7500 copies may hold a predictive value of recurrence in CC (AUC = 0.722; personal communication). Nevertheless, clinically oriented studies with long-term follow up are mandatory to validate these data.

The TTL has also been associated not only to the LN status, [26] but to different histological risk factors. In the present study, the presence of perineural and lymphatic invasion were independent factors related to TTL in both cohorts (p < 0.001; data not shown). In addition, in a recent multicenter study, our group showed a relationship of the TTL with size and pT stage in low grade tumors [45].

The study of multiple LNs at once reduces the molecular analysis to a single or few tests. This may help lessen some technical drawbacks such as the molecular evaluation of a large number of LNs separately, which may increase the likelihood of potential false positives. We obtained a much better overall accuracy with the H&E LN status in the pooling cohort than in the individual cohort. Additionally, the TTL of both cohorts did not statistically differ, and among pN0 cases, the TTLs were almost equal between them, and also with pN0 cases from other studies [26]. Thus, the pooling method may help to better assess the correct status of LNs in terms of LN positivity rates, as well as to provide reliable TTL data.

LN pooling also overcomes the second and most important drawback involved in performing molecular LN analysis in CRC, that is the amount of time invested in the analysis and the high costs of personnel and associated materials required. Patients’ TTL can be assessed with few molecular tests, analyzing at least the 12 LNs required. In our hands, the analysis was reduced to a median of only 2 molecular tests per case, and the time spent in the analysis was also greatly reduced, saving about 3 h of LN analysis per patient. The use of the pooling method enables the analysis of all LNs from 7.5 patients in the time needed to analyze one patient using the individual LN analysis. This represents a drastic drop of more than 85% of time and technical workload saved in molecular tests.

Our study has some drawbacks. Firstly, as stated, the two cohorts were not completely similar in terms of stage and tumor characteristics. In the individual cohort, cases with grossly positive LNs were discarded and pN1 corresponded to microscopic findings. Contrarily, the pooling cohort also included cases with gross positive LNs, as the inclusion criteria was made on the basis that fresh LN procurement could be performed. But although the pooling cohort had more high-grade cases and higher LN yields, it showed better correlation between the OSNA assay and the H&E evaluation of the LNs than the individual cohort. Secondly, the lack of follow-up of the pooling cohort makes it impracticable to interpret the significance of its results. Further studies are needed to obtain a predictive high-risk scale for stage I–II CC patients based on the TTL. As we lack the predictive values of the OSNA assay in colon cancer, pN staging is still mandatory, and LNs cannot be entirely submitted for molecular analysis.

Including in situ neoplasms and colon adenomas can be regarded as an additional drawback. However, there are some reports that these neoplasms may exceptionally harbor risk of metastatic disease [46, 47]. In addition, studies on circulating DNA have shown that in situ lesions may also harbor systemic disease in plasma [48, 49]. One of them included 85 patients (26 colorectal adenomas, 24 CRC and 35 normal patients). They found that adenomas had higher levels of total DNA, mitochondrial DNA, and Alu 247 bp fragment than normal controls, with a ROC curve of 0.797 [48]. Another study performed BEAMing PCR to detect mutant APC circulating DNA in patients with adenomas and metastatic and localized CRC and found that CRC had higher mutant APC DNA than controls. Nevertheless, 1 of the 11 adenomas had higher mutant APC DNA than controls [49]. They hypothesized that these fragments may derive from necrotic or apoptotic tumor cell DNA engulfed by macrophages and later released to de media [49]. Hence, the presence of low tumor burden detected by OSNA or other molecular techniques may not hold prognostic significance. For this reason, molecular analysis of LNs from precursor lesions and in situ neoplasms in our study could provide valuable data, as it may help to compare these lesions with early colon cancer, and to elucidate predictive values in this setting.

We believe that molecular LN staging in CRC performed with the OSNA analysis can replace the current pN histological staging in the near future. In order to be able to incorporate this new technology into the current pathologic staging, two conditions should be accomplished; firstly, to demonstrate that the OSNA results correlate with disease recurrence, with adequate positive and negative predictive values. Secondly, although the OSNA assay is easy, automated, fast, reproducible, and is already used in pathology departments for breast sentinel LN analysis, the high number of LNs to be analyzed in CCR, its high costs and time invested in molecular analysis were major drawbacks. In this study, we introduce the LN pooling approach with the OSNA assay to overcome these problems.

## Conclusions

In conclusion, our study shows that the OSNA pooling makes it possible to analyze a high number of LNs from surgical colectomies with few molecular tests per patient. It also enables quantification of the amount of tumor burden present in LNs, measured as the TTL. We propose the pooling strategy as a rapid method, which can be routinely performed in pathology departments. It enables the integration of more accurate molecular results into pathological LN staging. This would benefit the therapeutic management of patients.

## Methods

### Aim, design and setting

The aim of the study was to propose a feasible and efficient method that enables the integration of molecular diagnosis of colon cancer LN into pathology daily practice.

We describe the OSNA pooling technique as a laboratory procedure and compare the pooling and individual methods of OSNA assessment.

### Sample size and characteristics

This is an observational study performed at a single tertiary hospital. The Institutional Review Board approved the study, and informed consent was obtained from all patients. Patients’ demographics and specimen characteristics from both CC cohorts are described in Table 1.

#### Pooling cohort

All surgically resected colon neoplasms from February 2015 to December 2015 were included. Inclusion criteria were patients over 18 years old, with primary histologically confirmed colon carcinoma or adenoma with positive CK19 IHC. Exclusion criteria were rectal carcinomas, metastatic disease, familial adenomatous polyposis, inflammatory bowel disease or other malignancies, neoadjuvant therapy or stent-type intraluminal devices, extensive gross tumor infiltration of the mesocolon fat, and reception of the surgical specimen immersed in formalin. Eighty-six patients met the criteria and LN OSNA analysis was performed using the pooling method.

#### Individual cohort

Individual LN OSNA analysis was performed in 102 colectomies obtained from June 2012 to December 2013. Inclusion criteria were patients over 18 years old, with primary histologically confirmed colon carcinoma or adenoma with positive CK19 IHC. Exclusion criteria included rectal carcinomas, metastatic disease, synchronous tumors, cN1, familial adenomatous polyposis, inflammatory bowel disease or other malignancies, neo-adjuvant chemotherapy or stent-type intraluminal devices, presence of gross metastatic LN or gross tumor infiltration of the mesocolon fat, and reception of the surgical specimen immersed in formalin.

### Study procedure

All lymph nodes were analyzed by two methods, the conventional gold standard H&E and the OSNA assay.

#### Lymph node harvesting for H&E and deferred OSNA analysis

Lymph node dissection from all colectomy specimens was performed as described elsewhere [45]. Briefly, the mesocolon fat was detached from the colon wall, and dissection of the LNs from the mesocolon was performed on an ice-cooled and clean surface. In order to reproduce the standard LN H&E diagnosis, which it is usually performed by analyzing the central part of the LN, each LN was cut along the long axis. All LNs were analyzed by H&E and OSNA, irrespectively of their size. For larger LNs, a central 1 mm slice was put into a cassette for conventional formalin-fixation paraffin-embedding (FFPE) and H&E analysis. For small sized LNs, ½ of the LN was submitted for OSNA analysis and the other ½ for FFPE and H&E analysis. The rest of the LN was put into a microcentrifuge tube for delayed OSNA analysis (Fig. 1a, b). The same process was performed for all LN from the same patient.

At this point, depending on the cohort (individual or pooling), single or several LN were put into microcentrifuge tubes for ulterior OSNA analysis (Fig. 1c–e).

#### Individual OSNA lymph node analysis

Each dissected LN was put into separate microcentrifuge tubes for individual OSNA analysis. The tubes were stored at −80 °C until delayed OSNA analysis was performed (Fig. 1c).

#### Lymph node pooling for OSNA analysis

After dissection, LNs were put together into microcentrifuge tubes (pool). Each tube contained several LN up to 600 mg, which is the maximum loading weight of the OSNA assay. The tubes were then stored at −80 °C for delayed OSNA analysis (Fig. 1d, e).

#### H&E analysis

In both groups, a central 1 mm slice or ½ of the LN was FFPE for H&E analysis. In the individual group each LN was put into separate paraffin blocks for H&E diagnosis. In the pooling group, each paraffin block contained multiple LN (Fig. 1c, d).

### One-step nucleic acid amplification (OSNA) procedure and total tumor load (TTL)

Each microcentrifuge tube containing either an individual LN or a pool of LN was analyzed with the OSNA assay following the manufacturer’s instructions [24]. Briefly, harvested LN were homogenized with Lynorhag lysis buffer (Sysmex, Kobe, Japan), which stabilizes mRNA, protects against ribonucleases, and precipitates genomic DNA. After centrifugation, the homogenized product was analysed with the RD-100i system (Sysmex Corporation, Kobe, Japan), using the ready-to-use Lynoamp reaction kit (Sysmex, Kobe, Japan). The OSNA assay uses the RT-LAMP technology by which CK19 mRNA molecules are rapidly amplified without prior extraction or purification of RNA and/or DNA nucleic acids. The CK19 mRNA concentration is calculated indirectly via a byproduct (magnesium pyrophosphate) of the reaction [24, 40, 50, 51].

The results (CK19 mRNA copies/µL) were assessed in accordance with the cut-off determined in previous studies, considering a result of more than 250 copies/µL as positive [24, 28]. In the individual cohort the total tumor load (TTL) was defined as in breast cancer sentinel LN; i.e.; the sum of all the CK19 mRNA copies form all positive LNs in a given patient [27]. In the pooling cohort, the total tumor load of pools was defined as the sum of all CK19 mRNA copies from all the tubes analyzed per patient, which is the amount of tumor burden present in all LN dissected from a colectomy specimen, irrespective of it being positive or negative.

### Time invested in LN molecular analysis

Each run of the RD-100i system analyses up to 4 tubes simultaneously in 30–40 min, which includes preparation of the LN tissue: thawing, homogenization centrifugation, and 16 min of the RT-LAMP reaction.

### Pooling validation approach

**Fig. 2:**
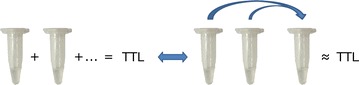
Pooling validation test. The OSNA result obtained from the sum of the different tubes containing pools (*left side*) of LN was equivalent to the OSNA result obtained from the analysis of a sample of the content of each tube and analyzed in a separate tube (*right side*)

In order to evaluate whether the final OSNA result obtained from the sum of each pool was equivalent to the result obtained from analyzing a mixture of the content of each of the pools in a separate tube (Fig. 2), we performed a validation assessment test. We used homogenized samples from 42/86 patients having their LN pooled in more than one tube (2–7 tubes). To simulate the pooling event, 20 µL of the homogenized sample of each of the pools was diluted with Lynorhag to a final volume of 200 µL and individually analyzed with OSNA (Fig. 2 left). In parallel, 20 µL of the homogenized sample of each of the tubes from a patient was put into a separate tube, diluted with Lynorhag to a final volume of 200 µL, and analyzed with OSNA (Fig. 2 right). The results of both procedures were compared. As in the regular OSNA assay, a result of ≥250 copies/µL was considered positive. Discordant results were analyzed twice (Table 3).

### CK19 immunohistochemistry

The OSNA assay is based on CK19 mRNA detection. CK19 IHC was performed on all primary colon carcinomas in order to exclude CK19 negative cases, not suitable to OSNA analysis. We used the primary CK19 antibody (CK19 mouse monoclonal, clone RCK108; IR615 pre-diluted. Dako, Denmark), as described in a previous study [45]. Positive IHC staining was considered when ≥10% of the cells presented membrane, with or without cytoplasmic staining.

### Pathology report and LN staging

Lymph node staging and pathology report were performed from the analysis of H&E stains, according to the American Joint Committee on Cancer protocol [52, 53]. The OSNA results were blind to the pathologist and clinician.

### Statistical analysis

Standard performance characteristics, including sensitivity, specificity, overall accuracy, positive predictive value (PPV) and negative predictive value (NPV), and their corresponding 95% confidence intervals (95% CI) were calculated to compare the OSNA assay with H&E. P-values of <0.05 were considered statistically significant. Statistical analysis was performed using SAS 9.2 (SAS Institute Inc., Cary, N.C.) for Windows. Cohen’s kappa coefficient for inter-rater agreement was calculated; with ≤0 indicating no agreement, 0.01–0.20 as none to slight, 0.21–0.40 as fair, 0.41–0.60 as moderate, 0.61–0.80 as substantial, and 0.81–1.00 as almost perfect agreement.

## Abbreviations

LN: lymph node
CC: colon cancer
TTL: total tumor load
CK19: cytokeratin 19
mRNA: messenger RNA
CRC: colorectal cancer
H&E: hematoxylin and eosin
OSNA: one step nucleic acid amplification
RT-LAMP: reverse transcription-loop mediated isothermal amplification
IHC: immunohistochemistry
FFPE: formalin-fixed paraffin-embedded
